# The Week's Work

**Published:** 1911-05-27

**Authors:** 


### The Week's Work.

### SOUTH MANCHESTER GUARDIANS AND THE ROYAL INFIRMARY.

The proposed grant of £50 to the Manchester Royal Infirmary by the above Guardians last week was made the occasion of a protest on grounds that, from their allegation against other institutions, time has made familiar. The objector, a lady, declared that the Guardians had no share in the management of the infirmary, and went on to allege that the poor patients were badly treated. Her one detailed instance was of a child with a broken arm, which, she said, was kept waiting for thirty-five minutes before it was attended to. The answer of the General Superintendent of the infirmary, Mr. W. G. Carnt, in an interview with the local press, is sufficiently comprehensive: "The accident room is never left. A doctor and nurses are always in attendance, and it is impossible for a patient to have to wait many minutes." The matter is worth noting, as on a vote being taken the lady's objection was not supported.

### OUR REPORT AND ITS SEQUEL AT COLCHESTER.

The Essex County Hospital is maintaining a hard struggle to prevent the lapse into debt from which it has escaped so recently. The Hospital may claim some share in assisting that struggle from the suggestions for increasing the income which were published on November 12, 1910, page 200, and from the account of the meeting where these suggestions were driven home which appeared in the same issue. The latest account of the hospital's doings extends only to December 31 last, and so there has not been time for much progress to be recorded, but we are glad to note that the Sunday Fund contribution at last has begun to rise to its old figure. Our suggestions for a linen league and for a workers' committee appear also to have been acted upon, but the formation of a Colchester Hospital Association does not seem to have been taken up. We shall look forward to the next annual report in the hope of finding that the increased support for which we have argued, and which the hospital certainly deserves, has been forthcoming from all these new sources.

### MEDICAL TEACHING IN BIRMINGHAM.

The staffs of the General and Queen's Hospitals, Birmingham, which hitherto have appointed a Board for the direction of the clinical teaching of the city, have come to an important arrangement with the University. In future the Clinical Board will be affiliated more closely to the University by the appointment of five academic representatives, each hospital now supplying two. The reconstituted Board will be responsible for all the details of the clinical teaching, but the University will administer the fees and the University staff will supply the teachers. Hitherto the Board of Education has declined to make a grant, refusing to recognise the medical course as technical, on the ground that the University was not wholly responsible for it. The new arrangement obviates this difficulty, and the medical subjects will now benefit accordingly.

### THE NEW GRADUATE HOSPITAL SCHOOL IN NEW YORK.

Work has been commenced on the new building for the New York Post-Graduate Medical School which is to cost 600,000 dollars and to provide four hundred beds. Several new features are to be introduced into the building; it will have a tower with rooms for fifty private patients, and on the roof of the tower will be a pavilion for open-air treatment. On three floors there is to be a loggia open to the street in front and to the rear, upon which beds can be kept permanently exposed to the air. In addition there will be three balconies to the eastward, and a roof-garden, so that plenty of provision is made for supplying patients with fresh air. There are to be eight small operating-rooms 18 feet by 20, but no large amphitheatre, as it is believed that the best results can be obtained by teaching small numbers of students at a time in close proximity to the operating-table. Laboratories are provided for research work and the study of tropical diseases. When the new building is completed the present quarters of the Post-Graduate School adjoining is to be rearranged and the nurses' home in connection with the institution is to be rebuilt at a cost of 100,000 dollars.

### A RECORD OF PROGRESS AT RUGBY.

The Hospital of St. Cross, Rugby, was the subject of a report in this journal on April 30, 1910. There Sir Henry Burdett remarked on the unsuitability of the nurses' accommodation, saying, "Much of it is mixed up with that for the maids, and, with the exception of three rooms it is as bad as bad can be." It is, therefore, interesting to learn from an interview with the matron of St. Cross Hospital, which the *Nursing Mirror* publishes this week, that a great effort has been made to secure enough money to obtain better rooms for nurses, a new laundry, and a servants' hall. Miss Osborne indeed remarks that "as soon as Mr. James (the hospital's president) read Sir Henry Burdett's comments in The Hospital on the nurses' rooms, he came to see them, and offered either to give us better ones or to supply the theatre. But we wanted the theatre more urgently." It is very gratifying to know how carefully the President of the St. Cross Hospital looks after its interests, but while congratulating it and him, it may perhaps be permissible to question whether it was really wise to place a new theatre before new nurses' quarters. The facts which influenced those who brought Mr. James round to their opinion are not before us, so we will say no more except that a tendency on occasion has been manifested all the country over to sacrifice the comfort of the nurses ostensibly to that of the patients, but in reality where a new theatre was in question to that of the surgeons. We understand that

£2,000, or one-third of the sum required for new nurses' quarters and a new laundry, has been received, and we hope that the nurses at any rate will be re-housed before the new laundry is begun. In any case these facts are interesting as another example to show that construction has followed and money been raised in the wake of Sir Henry's report.

### A CONTINENTAL CONTRIBUTION TO THE STUDY OF THE WARD UNIT.

Dr. G. van Eysselsteyn forwards us a copy of his paper on the ward unit ("*De Ziekenzaal cum adnexis*"—the sick ward with its annexes), of which a summary was originally published in *Het Ziekenhuis*. Detailed plans and criticism of the most modern ward designs are given, the criticism being in the main excellent. Dr. Goldwater's plan, lately published in The Hospital (February 4, 1911, pp. 563-4), comes in for a good deal of adverse comment, which seems fair in view of the criticism to which the design has been subjected by hospital experts both here and in America. Dr. van Eysselsteyn's ideal is the maximum of "light and fresh air everywhere, with the minimum of draught," and the plans given show various ingenious ways in which architects have attempted to attain to this ideal. The pamphlet, which is of great interest, may be obtained from the publishers, Messrs. F. Rossen, Amsterdam.

### THE LEAGUE OF MERCY GARDEN-PARTY AT WHITE LODGE.

A most enjoyable and successful garden-party on behalf of the League of Mercy was held on Wednesday afternoon in the charming grounds of the White Lodge. Lord and Lady Farquhar received their guests, who numbered nearly a thousand, at the entrance to the grounds and the representatives of the various districts in London and the Home Counties in which the League carries on its beneficent work were presented to the Grand President and Lady Grand President, Prince and Princess Alexander of Teck.

The King and Queen arrived shortly after 4.30 and once again exhibited their interest in the progress of the League and its work on behalf of the hospitals which their Majesties during the ten years they were Grand Presidents of the League invariably displayed. According to the charter under which the League of Mercy was founded in 1899, its object is to further the interests and promote the adequate maintenance of hospitals and other institutions for the relief of sickness and suffering, and this is effected especially by encouraging personal service on the part of large numbers of persons. Last year the League voted £18,000 to the King's Hospital Fund as against £1,000 in 1899, the first year of its operations. The League has, in the twelve years it has been in operation, contributed £153,000 to King Edward's Hospital Fund, in addition to which nearly £7,661 has been granted to local hospitals outside the area of the King's Fund. Last year £2,190 was awarded to local hospitals.

The work of the League is carried on by a network of some 100 "districts" spread over London and the Home Counties. Nearly every Parliamentary division within this area has its branch of the League, but even yet the organisation of its machinery is not fully completed. There are, however, 153 Presidents and Lady Presidents working under his Serene Highness Prince Alexander of Teck as Grand President and her Royal Highness Princess Alexander of Teck as Lady Grand President. Under these, and in co-operation with them, are some 1,200 ladies and gentlemen acting as Vice-Presidents and Lady Vice-Presidents and directing the work of some 20,000 members of the League, who in their turn have enrolled as associates, that is to say subscribers of from one shilling a year and upwards, more than 200,000 men, women, and children. Every shilling so subscribed goes direct to the hospitals without any deduction whatever. In this way the sympathy of a large army of the humbler citizens has been enlisted in the great and beneficent work of the hospitals, in the manner desired by his late Majesty King Edward when he inaugurated his Fund in the year of the Diamond Jubilee of her late Majesty Queen Victoria.

### HOSPITAL WORK IN AUSTRALIA.

The country hospitals in all parts of the Commonwealth, writes a Melbourne correspondent, have difficulty in getting and then in retaining trained nurses, although there are always many more applications from probationers than can be entertained. The Maryborough Hospital (Vic.) has been having particular difficulty. At a recent meeting the matron and a sister, the latter only three weeks on the staff, and the third to resign in six months, tendered their resignations (which were accepted). The sister explained that the nurses were kept at housework for several hours every morning and were not receiving proper training in nursing, for which they had entered the hospital, and "so far as I can see and gauge, the whole standard of the hospital is in a very lax condition." The Victorian State Treasurer (Mr. Watt) has been addressing himself to the problem of disparity of prices paid in different hospitals for the same articles, and has asked if it be not possible to standardise these essentials and for the medical charities to enter into joint contracts for their supply. The high cost of some of the supplies, as shown in the annual report of the Alfred Hospital, having attracted his attention, the annual Government grant is being temporarily withheld until explanations are forthcoming in the housing and treatment of the insane, the Government having decided to bring its asylums thoroughly up to date.

### THIS WEEK'S DRUG MARKET.

Business in drugs has been somewhat quieter again, but the prospects appear to be fairly good. As was anticipated, the price of mercury has been substantially reduced in price, and in consequence calomel, corrosive sublimate, and other salts of mercury are 1½d. per lb. cheaper. Cod-liver oil is again lower in value, and camphor also tends lower, but there is no quotable alteration in price. Jamaica sarsaparilla is rather dearer. The values of opium, morphine, and codeine are well maintained.

